# Crystal structure of (4b*S*,8a*R*)-1-isopropyl-4b,8,8-trimethyl-7-oxo-4b,7,8,8a,9,10-hexa­hydro­phenanthren-2-yl acetate

**DOI:** 10.1107/S2056989018005510

**Published:** 2018-04-27

**Authors:** Yassine Laamari, Moulay Youssef Ait Itto, Abdelkhalek Riahi, Sylviane Chevreux, Aziz Auhmani, El Mostafa Ketatni

**Affiliations:** a Laboratory of Organic Synthesis and Physico-Molecular Chemistry, Department of Chemistry, Faculty of Sciences Semlalia, BP 2390, Marrakech 40001, Morocco; b Institute of Molecular Chemistry of Reims, CNRS UMR 7312 Bat. Europol’Agro, Moulin of the Housse UFR Sciences, BP 1039-51687 Reims Cedex 2, France; c Laboratory of Applied Spectro-Chemistry and Environment, University Sultan Moulay Slimane, Faculty of Science and Technology, PO Box 523, Beni-Mellal, Morocco

**Keywords:** crystal structure, natural product, phenanthrene, C—H⋯O hydrogen bond, C—H⋯π inter­actions

## Abstract

The title compound, C_22_H_28_O_3_, was prepared by a direct acetyl­ation reaction of naturally occurring totarolenone. The mol­ecule contains three fused rings, which exhibit different conformations. The central ring has a half-chair conformation, while the non-aromatic oxo-substituted ring has a screw-boat conformation. In the crystal, mol­ecules are linked by C—H⋯O hydrogen bonds and C—H⋯π inter­actions, forming sheets parallel to the *bc* plane. The carbonyl O atoms and the C atom at the 6-position of the cyclo­hexene ring are each disordered over two sets of sites with major occupancy components of 0.63 (7) and 0.793 (14), respectively.

## Abstract 2

The title compound, (4b*S*,8a*R*)-1-isopropyl-4 b,8,8-trimethyl-7-oxo-4 b,7,8,8a,9,10-hexa­hydro­phenanthren-2-yl acetate, was prepared by a direct acetyl­ation reaction of naturally occurring totarolenone. In the crystal, mol­ecules are linked to each other by C—H⋯O hydrogen bonds and C—H⋯π inter­actions, forming sheets parallel to the *bc* plane.

## Chemical context   

Diterpene phenols are a family of natural products isolated from a variety of terrestrial plant sources. They exhibit a wide variety of inter­esting biological activities such as anti­tumour (Iwamoto *et al.*, 2003 ▸; Son *et al.* 2005 ▸), anti­microbial (Yoshikawa *et al.*, 2008 ▸; Pereda-Miranda *et al.*, 1992 ▸), anti­viral (Yang *et al.*, 2011 ▸; Wen *et al.*, 2007 ▸) and anti-inflammatory (Chen *et al.* 2013 ▸). In addition, derivatives of diterpene phenol natural products have been studied extensively as potential chemotherapeutic agents (Areche *et al.*, 2007 ▸; Yang *et al.* 2001 ▸).

**Figure 1:**
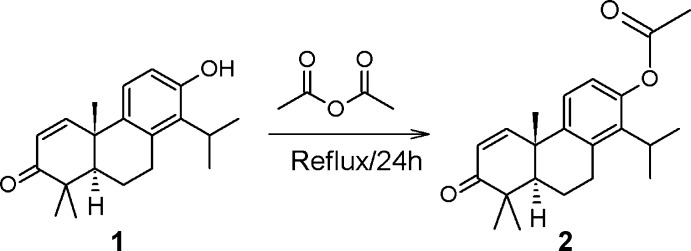
Reaction scheme for the synthesis of the title compound **2**.

With the aim of preparing diterpene phenol derivatives, we report here the hemisynthesis (Fig. 1 ▸) of (4b*S*,8a*R*)-1-isopropyl-4 b,8,8-trimethyl-7-oxo-4 b,7,8,8a,9,10-hexa­hydro­phenanthren-2-yl acetate, **2**, from naturally occurring totarolenone, **1**, extracted from the heartwood of *Tetra­clinis articulata* (Chow *et al.*, 1960 ▸). Treatment of **1** with acetic anhydride and pyridine provides compound **2** as colourless crystals in 88% yield. X-ray single crystal structure analysis allowed its structure to be confirmed unambiguously. Its structure was also characterized by ^1^H and ^13^C NMR measurements.
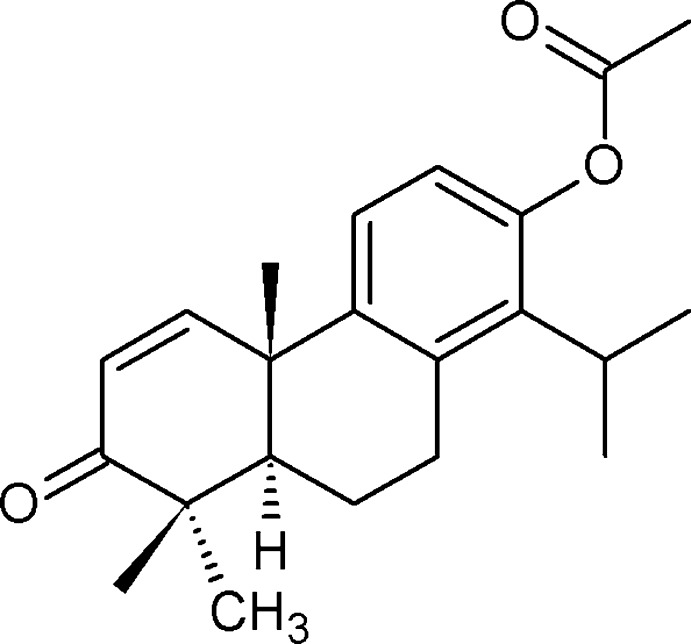


## Structural commentary   

**Figure 2:**
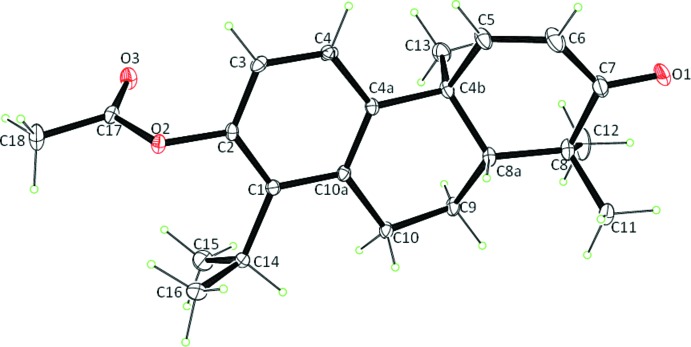
The mol­ecular structure of the title compound with the atom-numbering scheme. The displacement ellipsoids are drawn at the 30% probability level. Only the major disorder components are shown.

The mol­ecular structure is built up from three fused six-membered rings (Fig. 2 ▸). In the mol­ecule, there are two chiral carbon atoms, C4b exhibits an *S* configuration and C8a exhibits an *R* configuration. The central six-membered ring (C4*A*, C4*B*, C8*A*, C9, C10, C10*A*) assumes a half-chair conformation, as indicated by the total puckering amplitude *Q*_T_ = 0.55 (2) Å and spherical polar angle θ = 51.0 (2)° with φ = 136.0 (2)°. The major component of the cyclo­hexene ring exhibits a screw-boat conformation [*Q*_T_ = 0.462 (2) Å, θ =113.7 (2)°, φ = 145.9 (2)°] while the minor component has a chair conformation [*Q*_T_ = 0.558 (6) Å, θ =159.4 (6)°, φ = 235.6 (14)°].

## Supra­molecular features   

**Figure 3:**
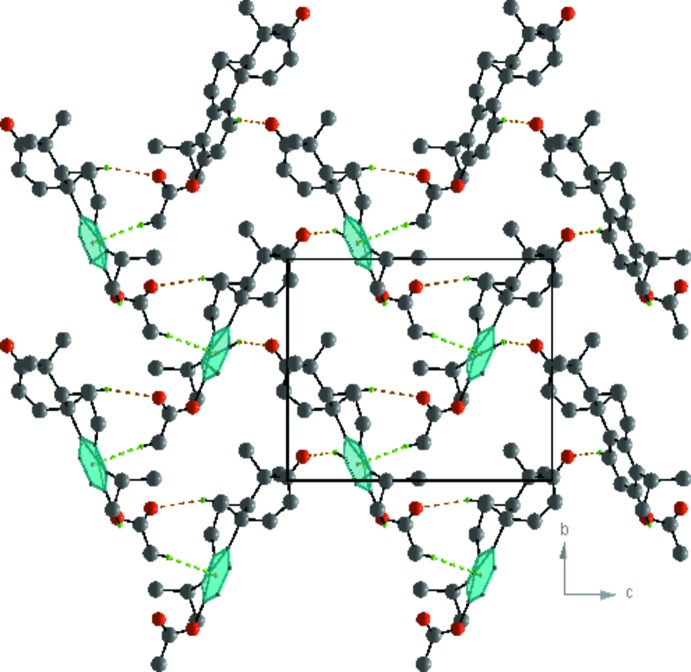
A view along the *a* axis of the crystal packing of the title compound, showing the C—H⋯O hydrogen bonds (orange dashed lines) and C—H⋯π inter­actions (green dashed lines). For clarity, only the H atoms involved in these inter­actions have been included.

In the crystal, mol­ecules are linked by C—H⋯O and C—H⋯π inter­actions, forming layers parallel to the *bc* plane (Table 1 ▸ and Fig. 3 ▸).

## Database survey   

**Figure 4:**
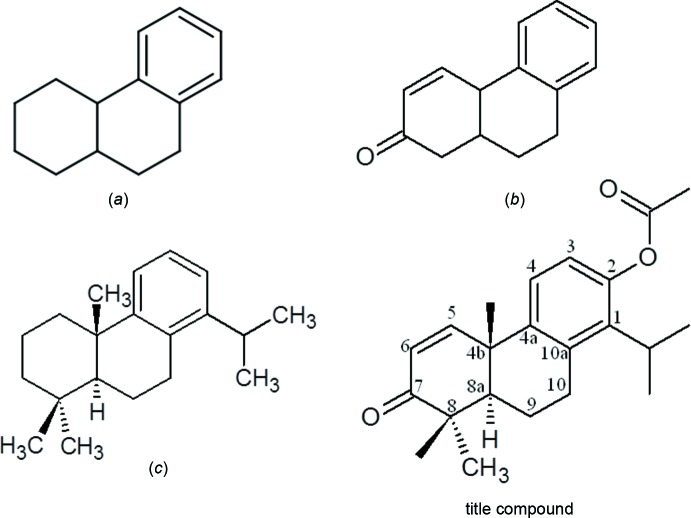
The core structures for database survey: (*a*) 1,2,3,4,4a,9,10,10*a*-hexa­hydro­phenanthren, and its (*b*) 7-oxo with double bond between C5 and C6, (*c*) 1-isopropyl-4 b,8,8-trimethyl substituents; and (*d*) the title compound.

A search of the Cambridge Structural Database using the 1,2,3,4,4a,9,10,10a-hexa­hydro­phenanthren ring system (Fig. 4 ▸ *a*) as the main skeleton, revealed the presence of 75 structures. These include several compounds similar to the title compound. One with a similar conformation has a hydroxyl substituent in place of the acetate in the title compound (Benharref *et al.*, 2011 ▸), and three others have a meth­oxy group in position 4b and carbaldehyde/benzene­sulfono­hydrazide (Vo *et al.*, 2008 ▸) or bi­phenyl­sulfonyl (Gu & You, 2011 ▸) in position 9 (Fig. 4 ▸ *b*), while six entries (Oubabi *et al.*, 2014*a* ▸,*b* ▸; Zeroual *et al.*, 2007 ▸, 2008 ▸; Cutfield *et al.*, 1974 ▸; Pettit *et al.*, 2004 ▸) have 1-isopropyl-4b,8,8-trimethyl substit­uents (Fig. 4 ▸ *c*).

## Synthesis and crystallization   

A solution of totarolenone **1** (300 mg, 1.041 mmol) in acetic anhydride (10 mL) and sodium acetate (290 mg) was heated under reflux for 24 h. After cooling, the solution was extracted with ether (3 × 20 mL). The organic layer was washed with water, dried on anhydrous Na_2_SO_4_ and evaporated under reduced pressure. The obtained residue was chromatographed on silica gel column using hexane and ethyl acetate (95/5) as eluent, to give compound **2**.

## Refinement   

Crystal data, data collection and structure refinement details are summarized in Table 2 ▸. H atoms were placed in calculated positions and refined in the riding model: C—H = 0.95–1.00 Å with *U*_iso_(H) = 1.2*U*_eq_(C) or 1.5*U*_eq_(C-methyl). The carbonyl O atom is disordered over two sites having occupancies of 0.63 (7) and 0.37 (7). Atom C6 atom of the cyclo­hexene ring is disordered over two sites with an occupancy ratio of 0.793 (14):0.207 (14). The absolute structure was reliably determined based on the value of the Flack parameter [0.02 (5)].

## Supplementary Material

Crystal structure: contains datablock(s) I, global. DOI: 10.1107/S2056989018005510/xu5922sup1.cif

Structure factors: contains datablock(s) I. DOI: 10.1107/S2056989018005510/xu5922Isup2.hkl

CCDC reference: 1835842

Additional supporting information:  crystallographic information; 3D view; checkCIF report

## Figures and Tables

**Table 1:** Hydrogen-bond geometry (Å, °) *Cg*1 is the centroid of the benzene ring.

*D*—H⋯*A*	*D*—H	H⋯*A*	*D*⋯*A*	*D*—H⋯*A*
C4—H4⋯O1^i^	0.95	2.50	3.411 (10)	159
C4—H4⋯O1*A* ^i^	0.95	2.55	3.428 (17)	154
C10—H10*B*⋯O1*A* ^ii^	0.99	2.55	3.328 (17)	136
C13—H13*C*⋯O3^iii^	0.98	2.59	3.530 (2)	161
C18—H18*A*⋯*Cg*1^iv^	0.98	2.55	3.504 (2)	165

Symmetry codes: (i) 
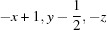
; (ii) 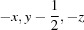; (iii) 
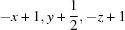
; (iv) 
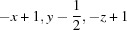
.

**Table 2:** Experimental details

Crystal data	
Chemical formula	C_22_H_28_O_3_
*M*_r_	340.44
Crystal system, space group	Monoclinic, *P*2_1_
Temperature (K)	100
*a*, *b*, *c* (Å)	7.4103 (2), 10.4681 (3), 12.8121 (3)
β (°)	102.235 (1)
*V* (Å^3^)	971.28 (4)
*Z*	2
Radiation type	Cu *K*α
μ (mm^−1^)	0.60
Crystal size (mm)	0.41 × 0.30 × 0.18

Data collection	
Diffractometer	D8 Venture CMOS area detector
Absorption correction	Numerical (*SADABS*; Bruker, 2012 ▸)
No. of measured, independent and observed [*I* > 2σ(*I*)] reflections	17946, 3909, 3852
*R*_int_	0.029
(sin θ/λ)_max_ (Å^−1^)	0.625

Refinement	
*R*[*F* ^2^ > 2σ(*F* ^2^)], *wR*(*F* ^2^), *S*	0.030, 0.077, 1.05
No. of reflections	3909
No. of parameters	252
No. of restraints	1
H-atom treatment	H-atom parameters constrained
Δρ_max_, Δρ_min_ (e Å^−3^)	0.16, −0.17
Absolute structure	Flack *x* determined using 1757 quotients [(*I* ^+^)−(*I* ^−^)]/[(*I* ^+^)+(*I* ^−^)] (Parsons *et al.*, 2013 ▸)
Absolute structure parameter	0.02 (5)

Computer programs: *APEX2* and *SAINT* (Bruker, 2012 ▸), *SHELXS2014* (Sheldrick, 2008 ▸), *SHELXL2014* (Sheldrick, 2015 ▸), *ORTEP-3 for Windows* (Farrugia, 2012 ▸), *DIAMOND* (Brandenburg *et al.*, 2012 ▸), *PLATON* (Spek, 2009 ▸) and *publCIF* (Westrip, 2010 ▸).

## supplementary crystallographic information

### Crystal data

**Table:**

C_22_H_28_O_3_	*F*(000) = 368
*M**_r_* = 340.44	*D*_x_ = 1.164 Mg m^−^^3^
Monoclinic, *P*2_1_	Cu *K*α radiation, λ = 1.54178 Å
*a* = 7.4103 (2) Å	Cell parameters from 3909 reflections
*b* = 10.4681 (3) Å	θ = 3.5–74.5°
*c* = 12.8121 (3) Å	µ = 0.60 mm^−^^1^
β = 102.235 (1)°	*T* = 100 K
*V* = 971.28 (4) Å^3^	Block, colourless
*Z* = 2	0.41 × 0.30 × 0.18 mm

### Data collection

**Table:**

D8 Venture CMOS area detector diffractometer	3852 reflections with *I* > 2σ(*I*)
Radiation source: microsource	*R*_int_ = 0.029
φ and ω scans	θ_max_ = 74.5°, θ_min_ = 3.5°
Absorption correction: numerical (SADABS; Bruker, 2012)	*h* = −9→9
	*k* = −13→13
17946 measured reflections	*l* = −16→15
3909 independent reflections	

### Refinement

**Table:**

Refinement on *F*^2^	Hydrogen site location: inferred from neighbouring sites
Least-squares matrix: full	H-atom parameters constrained
*R*[*F*^2^ > 2σ(*F*^2^)] = 0.030	*w* = 1/[σ^2^(*F*_o_^2^) + (0.0376*P*)^2^ + 0.2106*P*] where *P* = (*F*_o_^2^ + 2*F*_c_^2^)/3
*wR*(*F*^2^) = 0.077	(Δ/σ)_max_ < 0.001
*S* = 1.05	Δρ_max_ = 0.16 e Å^−^^3^
3909 reflections	Δρ_min_ = −0.17 e Å^−^^3^
252 parameters	Absolute structure: Flack *x* determined using 1757 quotients [(*I*^+^)-(*I*^-^)]/[(*I*^+^)+(*I*^-^)] (Parsons *et al.*, 2013)
1 restraint	Absolute structure parameter: 0.02 (5)

### Special details

**Table:**

Geometry. All esds (except the esd in the dihedral angle between two l.s. planes) are estimated using the full covariance matrix. The cell esds are taken into account individually in the estimation of esds in distances, angles and torsion angles; correlations between esds in cell parameters are only used when they are defined by crystal symmetry. An approximate (isotropic) treatment of cell esds is used for estimating esds involving l.s. planes.

### Fractional atomic coordinates and isotropic or equivalent isotropic displacement parameters (Å^2^)

**Table:**

	*x*	*y*	*z*	*U*_iso_*/*U*_eq_	Occ. (<1)
O2	0.31864 (16)	−0.17015 (11)	0.35741 (9)	0.0169 (2)	
O3	0.57128 (17)	−0.12685 (12)	0.48549 (10)	0.0228 (3)	
C1	0.1762 (2)	0.03631 (15)	0.31947 (13)	0.0149 (3)	
C2	0.3194 (2)	−0.04717 (15)	0.31317 (12)	0.0141 (3)	
C3	0.4591 (2)	−0.01616 (16)	0.26178 (12)	0.0165 (3)	
H3	0.5532	−0.0764	0.2575	0.020*	
C4	0.4606 (2)	0.10335 (17)	0.21673 (12)	0.0160 (3)	
H4	0.5571	0.1253	0.1817	0.019*	
C4A	0.3228 (2)	0.19262 (15)	0.22171 (12)	0.0140 (3)	
C4B	0.3352 (2)	0.32561 (16)	0.17177 (13)	0.0167 (3)	
C5	0.3964 (3)	0.31576 (18)	0.06569 (14)	0.0242 (4)	
H5	0.4747	0.2476	0.0544	0.029*	
C7	0.2283 (3)	0.5140 (2)	−0.00216 (14)	0.0262 (4)	
C8	0.1175 (3)	0.51452 (17)	0.08559 (13)	0.0217 (4)	
C8A	0.1410 (2)	0.38675 (17)	0.14900 (12)	0.0166 (3)	
H8A	0.0595	0.3243	0.1021	0.020*	
C9	0.0693 (2)	0.39088 (17)	0.25247 (13)	0.0195 (3)	
H9A	0.1618	0.4325	0.3095	0.023*	
H9B	−0.0461	0.4415	0.2413	0.023*	
C10	0.0326 (2)	0.25550 (16)	0.28606 (13)	0.0181 (3)	
H10A	0.0204	0.2569	0.3615	0.022*	
H10B	−0.0866	0.2260	0.2423	0.022*	
C10A	0.1817 (2)	0.16011 (16)	0.27480 (12)	0.0143 (3)	
C11	−0.0867 (3)	0.5279 (2)	0.02894 (17)	0.0308 (4)	
H11A	−0.1038	0.6076	−0.0122	0.046*	
H11B	−0.1639	0.5294	0.0823	0.046*	
H11C	−0.1223	0.4554	−0.0194	0.046*	
C12	0.1723 (4)	0.63433 (19)	0.15482 (19)	0.0409 (6)	
H12A	0.1463	0.7108	0.1101	0.061*	
H12B	0.3044	0.6310	0.1874	0.061*	
H12C	0.1011	0.6374	0.2111	0.061*	
C13	0.4868 (3)	0.40095 (19)	0.24898 (16)	0.0252 (4)	
H13A	0.5014	0.4854	0.2188	0.038*	
H13B	0.6039	0.3543	0.2592	0.038*	
H13C	0.4511	0.4109	0.3180	0.038*	
C14	0.0212 (2)	−0.00178 (18)	0.37495 (14)	0.0220 (4)	
H14	−0.0789	0.0631	0.3538	0.026*	
C15	0.0844 (3)	0.0071 (3)	0.49646 (16)	0.0347 (5)	
H15A	0.1850	−0.0538	0.5209	0.052*	
H15B	−0.0193	−0.0132	0.5300	0.052*	
H15C	0.1280	0.0939	0.5163	0.052*	
C16	−0.0662 (3)	−0.1325 (2)	0.3410 (2)	0.0352 (5)	
H16A	−0.0992	−0.1373	0.2629	0.053*	
H16B	−0.1774	−0.1433	0.3701	0.053*	
H16C	0.0224	−0.2003	0.3685	0.053*	
C17	0.4501 (2)	−0.19878 (16)	0.44565 (13)	0.0165 (3)	
C18	0.4186 (3)	−0.32928 (18)	0.48463 (16)	0.0285 (4)	
H18A	0.5095	−0.3469	0.5506	0.043*	
H18B	0.4316	−0.3924	0.4302	0.043*	
H18C	0.2939	−0.3344	0.4988	0.043*	
C6	0.3407 (7)	0.4028 (4)	−0.0124 (3)	0.0322 (13)	0.793 (14)
H6	0.3774	0.3905	−0.0784	0.039*	0.793 (14)
C6A	0.4227 (16)	0.4506 (11)	0.0182 (8)	0.014 (3)	0.207 (14)
H6A	0.5335	0.4861	0.0047	0.017*	0.207 (14)
O1	0.222 (3)	0.6074 (7)	−0.0588 (15)	0.040 (3)	0.63 (7)
O1A	0.185 (3)	0.575 (6)	−0.084 (3)	0.059 (7)	0.37 (7)

### Atomic displacement parameters (Å^2^)

**Table:**

	*U*^11^	*U*^22^	*U*^33^	*U*^12^	*U*^13^	*U*^23^
O2	0.0188 (5)	0.0125 (5)	0.0173 (5)	−0.0001 (4)	−0.0009 (4)	0.0017 (4)
O3	0.0257 (6)	0.0197 (6)	0.0195 (6)	−0.0038 (5)	−0.0032 (5)	0.0020 (5)
C1	0.0147 (7)	0.0179 (8)	0.0122 (7)	0.0008 (6)	0.0026 (6)	0.0017 (6)
C2	0.0173 (8)	0.0117 (7)	0.0116 (7)	−0.0003 (6)	−0.0006 (6)	0.0008 (6)
C3	0.0167 (7)	0.0189 (8)	0.0139 (7)	0.0058 (6)	0.0028 (6)	−0.0016 (6)
C4	0.0153 (8)	0.0205 (8)	0.0131 (7)	0.0004 (6)	0.0050 (6)	−0.0002 (6)
C4A	0.0182 (8)	0.0139 (7)	0.0095 (7)	0.0002 (6)	0.0022 (6)	0.0000 (6)
C4B	0.0233 (8)	0.0145 (7)	0.0135 (7)	−0.0003 (6)	0.0067 (6)	0.0014 (6)
C5	0.0375 (10)	0.0191 (8)	0.0201 (8)	0.0019 (8)	0.0153 (7)	0.0021 (7)
C7	0.0286 (9)	0.0265 (9)	0.0229 (8)	−0.0030 (8)	0.0040 (7)	0.0099 (8)
C8	0.0302 (9)	0.0153 (8)	0.0193 (8)	0.0023 (7)	0.0043 (7)	0.0048 (7)
C8A	0.0248 (8)	0.0129 (7)	0.0124 (7)	0.0018 (6)	0.0043 (6)	0.0017 (6)
C9	0.0277 (9)	0.0150 (7)	0.0178 (8)	0.0080 (7)	0.0095 (6)	0.0019 (6)
C10	0.0223 (8)	0.0177 (8)	0.0166 (8)	0.0066 (6)	0.0093 (6)	0.0047 (6)
C10A	0.0173 (7)	0.0152 (7)	0.0105 (7)	0.0031 (6)	0.0030 (6)	0.0009 (5)
C11	0.0303 (10)	0.0309 (10)	0.0317 (10)	0.0100 (8)	0.0074 (8)	0.0162 (8)
C12	0.0713 (17)	0.0125 (9)	0.0354 (11)	0.0019 (9)	0.0030 (11)	0.0015 (8)
C13	0.0272 (9)	0.0194 (8)	0.0287 (9)	−0.0055 (7)	0.0053 (7)	0.0001 (7)
C14	0.0174 (8)	0.0239 (9)	0.0272 (9)	0.0060 (7)	0.0103 (7)	0.0119 (7)
C15	0.0367 (11)	0.0482 (12)	0.0249 (9)	0.0116 (10)	0.0192 (8)	0.0138 (9)
C16	0.0174 (8)	0.0298 (11)	0.0587 (13)	−0.0018 (8)	0.0084 (8)	0.0144 (10)
C17	0.0203 (8)	0.0149 (8)	0.0139 (7)	0.0044 (6)	0.0025 (6)	0.0001 (6)
C18	0.0363 (11)	0.0171 (9)	0.0273 (10)	−0.0016 (8)	−0.0037 (8)	0.0071 (7)
C6	0.048 (3)	0.0312 (18)	0.0220 (14)	0.0010 (19)	0.0181 (16)	0.0045 (13)
C6A	0.014 (5)	0.016 (5)	0.014 (4)	−0.010 (4)	0.004 (4)	0.001 (4)
O1	0.042 (5)	0.033 (3)	0.051 (4)	0.010 (2)	0.026 (3)	0.028 (2)
O1A	0.033 (5)	0.095 (17)	0.048 (8)	0.014 (7)	0.012 (5)	0.055 (9)

### Geometric parameters (Å, º)

**Table:**

O2—C17	1.360 (2)	C9—H9A	0.9900
O2—C2	1.4071 (19)	C9—H9B	0.9900
O3—C17	1.200 (2)	C10—C10A	1.519 (2)
C1—C2	1.390 (2)	C10—H10A	0.9900
C1—C10A	1.421 (2)	C10—H10B	0.9900
C1—C14	1.526 (2)	C11—H11A	0.9800
C2—C3	1.378 (2)	C11—H11B	0.9800
C3—C4	1.379 (2)	C11—H11C	0.9800
C3—H3	0.9500	C12—H12A	0.9800
C4—C4A	1.396 (2)	C12—H12B	0.9800
C4—H4	0.9500	C12—H12C	0.9800
C4A—C10A	1.404 (2)	C13—H13A	0.9800
C4A—C4B	1.543 (2)	C13—H13B	0.9800
C4B—C5	1.525 (2)	C13—H13C	0.9800
C4B—C8A	1.545 (2)	C14—C15	1.531 (3)
C4B—C13	1.546 (2)	C14—C16	1.537 (3)
C5—C6	1.352 (3)	C14—H14	1.0000
C5—C6A	1.565 (11)	C15—H15A	0.9800
C5—H5	0.9500	C15—H15B	0.9800
C7—O1	1.213 (10)	C15—H15C	0.9800
C7—O1A	1.215 (16)	C16—H16A	0.9800
C7—C6	1.453 (4)	C16—H16B	0.9800
C7—C8	1.526 (3)	C16—H16C	0.9800
C7—C6A	1.557 (9)	C17—C18	1.490 (2)
C8—C11	1.540 (3)	C18—H18A	0.9800
C8—C12	1.540 (3)	C18—H18B	0.9800
C8—C8A	1.556 (2)	C18—H18C	0.9800
C8A—C9	1.530 (2)	C6—H6	0.9500
C8A—H8A	1.0000	C6A—H6A	0.9500
C9—C10	1.522 (2)		
			
C17—O2—C2	118.24 (12)	C4A—C10A—C1	120.28 (14)
C2—C1—C10A	117.54 (14)	C4A—C10A—C10	121.20 (14)
C2—C1—C14	121.45 (14)	C1—C10A—C10	118.53 (14)
C10A—C1—C14	120.98 (14)	C8—C11—H11A	109.5
C3—C2—C1	122.65 (15)	C8—C11—H11B	109.5
C3—C2—O2	118.38 (14)	H11A—C11—H11B	109.5
C1—C2—O2	118.92 (14)	C8—C11—H11C	109.5
C2—C3—C4	119.16 (15)	H11A—C11—H11C	109.5
C2—C3—H3	120.4	H11B—C11—H11C	109.5
C4—C3—H3	120.4	C8—C12—H12A	109.5
C3—C4—C4A	121.16 (15)	C8—C12—H12B	109.5
C3—C4—H4	119.4	H12A—C12—H12B	109.5
C4A—C4—H4	119.4	C8—C12—H12C	109.5
C4—C4A—C10A	119.13 (14)	H12A—C12—H12C	109.5
C4—C4A—C4B	118.45 (14)	H12B—C12—H12C	109.5
C10A—C4A—C4B	122.38 (14)	C4B—C13—H13A	109.5
C5—C4B—C4A	111.34 (13)	C4B—C13—H13B	109.5
C5—C4B—C8A	107.50 (13)	H13A—C13—H13B	109.5
C4A—C4B—C8A	108.47 (13)	C4B—C13—H13C	109.5
C5—C4B—C13	106.98 (14)	H13A—C13—H13C	109.5
C4A—C4B—C13	107.10 (13)	H13B—C13—H13C	109.5
C8A—C4B—C13	115.50 (14)	C1—C14—C15	111.07 (15)
C6—C5—C4B	120.85 (18)	C1—C14—C16	114.48 (16)
C4B—C5—C6A	111.8 (4)	C15—C14—C16	111.19 (17)
C6—C5—H5	119.6	C1—C14—H14	106.5
C4B—C5—H5	119.6	C15—C14—H14	106.5
O1—C7—C8	118.7 (5)	C16—C14—H14	106.5
O1A—C7—C8	123.5 (13)	C14—C15—H15A	109.5
C6—C7—C8	118.54 (17)	C14—C15—H15B	109.5
C8—C7—C6A	120.1 (3)	H15A—C15—H15B	109.5
C7—C8—C11	106.34 (15)	C14—C15—H15C	109.5
C7—C8—C12	108.06 (17)	H15A—C15—H15C	109.5
C11—C8—C12	108.06 (18)	H15B—C15—H15C	109.5
C7—C8—C8A	111.32 (15)	C14—C16—H16A	109.5
C11—C8—C8A	108.33 (15)	C14—C16—H16B	109.5
C12—C8—C8A	114.38 (14)	H16A—C16—H16B	109.5
C9—C8A—C4B	109.20 (13)	C14—C16—H16C	109.5
C9—C8A—C8	114.04 (14)	H16A—C16—H16C	109.5
C4B—C8A—C8	116.83 (14)	H16B—C16—H16C	109.5
C9—C8A—H8A	105.2	O3—C17—O2	123.70 (15)
C4B—C8A—H8A	105.2	O3—C17—C18	126.10 (15)
C8—C8A—H8A	105.2	O2—C17—C18	110.19 (14)
C10—C9—C8A	109.53 (14)	C17—C18—H18A	109.5
C10—C9—H9A	109.8	C17—C18—H18B	109.5
C8A—C9—H9A	109.8	H18A—C18—H18B	109.5
C10—C9—H9B	109.8	C17—C18—H18C	109.5
C8A—C9—H9B	109.8	H18A—C18—H18C	109.5
H9A—C9—H9B	108.2	H18B—C18—H18C	109.5
C10A—C10—C9	114.07 (14)	C5—C6—C7	124.3 (2)
C10A—C10—H10A	108.7	C5—C6—H6	117.9
C9—C10—H10A	108.7	C7—C6—H6	117.9
C10A—C10—H10B	108.7	C7—C6A—C5	105.2 (7)
C9—C10—H10B	108.7	C7—C6A—H6A	127.4
H10A—C10—H10B	107.6	C5—C6A—H6A	127.4

### Hydrogen-bond geometry (Å, º)

Cg1 is the centroid of the benzene ring.

**Table:**

*D*—H···*A*	*D*—H	H···*A*	*D*···*A*	*D*—H···*A*
C4—H4···O1^i^	0.95	2.50	3.411 (10)	159
C4—H4···O1*A*^i^	0.95	2.55	3.428 (17)	154
C10—H10*B*···O1*A*^ii^	0.99	2.55	3.328 (17)	136
C13—H13*C*···O3^iii^	0.98	2.59	3.530 (2)	161
C18—H18*A*···*Cg*1^iv^	0.98	2.55	3.504 (2)	165

Symmetry codes: (i) −*x*+1, *y*−1/2, −*z*; (ii) −*x*, *y*−1/2, −*z*; (iii) −*x*+1, *y*+1/2, −*z*+1; (iv) −*x*+1, *y*−1/2, −*z*+1.
